# Barriers and facilitators of domain-specific physical activity: a systematic review of reviews

**DOI:** 10.1186/s12889-022-14385-1

**Published:** 2022-10-26

**Authors:** Leandro Garcia, Gerfeson Mendonça, Tânia R. Bertoldo Benedetti, Lucélia Justino Borges, Inês Amanda Streit, Marina Christofoletti, Fernando Lopes e Silva-Júnior, Camila Bosquiero Papini, Maria Angélica Binotto

**Affiliations:** 1 Centre for Public Health, Queen’s University Belfast, Institute of Clinical Sciences, Royal Victoria Hospital, Belfast, BT12 6BA UK; 2 Federal University of Alagoas, Maceió, Alagoas Brazil; 3 CESMAC University Centre, Maceió, Alagoas Brazil; 4 Department of Physical Education, Federal University of Santa Catarina, Florianópolis, Santa Catarina Brazil; 5 Department of Physical Education, Federal University of Paraná, Curitiba, Paraná Brazil; 6 Federal University of Amazonas, Manaus, Amazonas Brazil; 7 School of Medicine, Federal University of Piauí, Teresina, Piauí Brazil; 8 Department of Sports Science, Federal University of the Triângulo Mineiro, Uberaba, Minas Gerais Brazil; 9 Department of Physical Education, State University of the Centro-Oeste, Irati, Paraná Brazil

**Keywords:** Physical activity, Exercise, Determinants, Correlates, Predictors, Built environment, Social environment, Psychological factors, Umbrella review, Literature review

## Abstract

**Background:**

Knowing what facilitates and hinders physical activity behaviour across domains (leisure, travel, work or education, and household) is central for the development of actions for more active lifestyles. Thus, the aim of this systematic review of reviews was to summarize the evidence on barriers and facilitators of domain-specific physical activity.

**Methods:**

We included systematic reviews with or without meta-analysis that investigated the association between modifiable barriers and facilitators and levels of domain-specific physical activity. Reviews published until September 2020 were retrieved from PubMed, ISI Web of Science, Scopus, Regional Library of Medicine (BIREME), and PsycNET, and from the reference list of selected articles. Each review was screened by two independent reviewers for eligibility. Data extracted from selected papers included methodological aspects (number of primary studies, study designs, and age groups); physical activity domains and barriers and facilitators investigated; and direction of association. For each pair of barrier/facilitator and domain-specific physical activity, we recorded the number of positive, negative, and null associations reported across reviews. Quality assessment of each systematic review was performed using the AMSTAR-2 tool.

**Results:**

Forty-four systematic reviews were selected. The evidence base was largest for leisure-time followed by travel-related physical activity. A very small number of reviews included physical activity in work, educational and domestic settings. Across all physical activity domains, factors related to the built environment were more abundant in the reviews than intra and interpersonal factors. Very consistent positive associations were observed between a range of intrapersonal factors and leisure-time physical activity, as well as moderately consistent evidence of positive association for general social support and support from family members. Evidence of moderate consistency was found for the positive association between transport-related physical activity and positive beliefs about consequences, walkability, and existence of facilities that support active travel. Evidence on barriers and facilitators for physical activity at work, educational, and domestic settings was limited in volume and consistency.

**Conclusions:**

Efforts and resources are required to diversify and strength the evidence base on barriers and facilitators of domain-specific physical activity, as it is still limited and biased towards the leisure domain and built environment factors.

**Trial registration:**

PROSPERO CRD42020209710.

**Supplementary Information:**

The online version contains supplementary material available at 10.1186/s12889-022-14385-1.

## Background

The health benefits of physical activity are well established [1]. However, global progress to increasing physical activity has been slow [2]. Worldwide, 27.5% of adults [3] and 81% of adolescents [4] do not meet the recommended levels of physical activity. Physical activity can be undertaken in different domains, named leisure, travel, work or education, and household [1]. These domains reflect when, where, and how physical activity is performed according to the routine of daily living, opportunities, duties, and culture. For instance, a significant fraction of the volume of moderate-to-vigorous physical activity comes from occupational and household activities (less volitional domains) [5]. In contrast, the recreational domain (most volitional domain) usually contributes the least to the total physical activity volume [5].

There is some evidence that physical activity performed in different domains may have different effects on health [6, 7]. For instance, harmful health outcomes have been associated with high levels of occupational physical activity, with an 18% increase in the risk of premature mortality compared to those in less physically demanding jobs [7]. The effects of physical activity on mental health may also vary according to domain, with one meta-analysis indicating that, compared with other domains, recreational physical activity could be more effective in preventing ill mental health than other domains [8].

A number of intrapersonal, interpersonal, environmental, cultural, socio-economic, and political factors can influence individual and population patterns of physical activity [9, 10]. However, the direction and magnitude of the relationship between these factors and physical activity level may depend on the physical activity domain (leisure, travel, work or education, and household) of interest. Mitigating barriers and strengthening facilitators in the different domains is key to enable the adoption and maintenance of a more active lifestyle for all. Therefore, evidence on domain-specific barriers and facilitators becomes central to the development of more effective physical activity promotion actions. Thus, our objective was to conduct a systematic review of reviews on barriers and facilitators of domain-specific physical activity.

## Methods

We conducted a systematic review of reviews following the Preferred Reporting Items for Systematic Reviews and Meta-analysis (PRISMA) guidelines [11]. The study protocol was registered and approved in the International Prospective Register of Systematic Reviews (PROSPERO) under the code CRD42020209710.

### Definition of terms

We defined barriers as factors that hinder, limit, or prevent people from engaging in a certain behaviour, whereas facilitators are factors that favour, facilitate, or help people to engage in a certain behaviour. Only potentially modifiable barriers and facilitators were included in this review, such as lack of time, attitude, motivation, aspects of the perceived and built environment, and social support from friends and family.

### Eligibility criteria

Table 1 presents the eligibility criteria according to participants, exposure, comparators, outcomes and study design [11]. We included systematic reviews with or without meta-analysis that investigated at least one of the physical activity domains (leisure, travel, work or education, or household), published from the inception of the reference databases until September 2020 (date of search). No limits were imposed on the type of original studies included by the reviews (e.g., quantitative, qualitative, or mixed methods), as well as age group investigated, date or geographic location of the original studies.

**Table 1:** Eligibility criteria

Include	Exclude
**Participants**	
Human participants. No restrictions on participants’ attributes (e.g., age, sex, disability, socio-economic status)	
**Exposure**	
Greater exposure to potentially modifiable barriers or facilitators of domain-specific physical activity (as defined in Definition of terms)	Non-modifiable factors, such as demographic attributes, weather, and terrain
**Comparators**	
Lower exposure to potentially modifiable barriers or facilitators of domain-specific physical activity	
**Outcomes**	
Self-reported or device-measured engagement in, or greater volume of, domain-specific physical activity (leisure, travel, work or education, or household)	Measures that combine physical activity domains or that combine domain-specific physical activity with other behaviours or risk factors
**Study design**	
Peer-reviewed systematic reviews articles with or without meta-analysis. No restrictions on the methodological approach of original studies included by the reviews (e.g., quantitative, qualitative, or mixed methods)	Absence of complete description of methods and results (e.g., short articles, conference abstracts). Theses, dissertations, points of view, essays, and editorials. Articles published in languages other than English, Spanish, or Portuguese (languages spoken by the review team)

We excluded review articles that were not peer-reviewed, did not provide a complete description of methods and results (e.g., short articles, conference abstracts), did not investigate potentially modifiable factors (see details in Definition of terms), or were not published in English, Spanish, or Portuguese (languages spoken by the review team).

### Search and study selection

Searches were performed in PubMed, ISI Web of Science, Scopus, Regional Library of Medicine (BIREME), and PsycNET. In addition, the reference list of the included studies was consulted.

The electronic search strategy used key terms aligned with the pre-established eligibility criteria in Table 1. Database-specific indexing terms (e.g., MeSH terms) and free-text words were combined using the boolean operators “AND” and “OR”. See Additional File 1 for search string used in each database.

**Fig. 1:**
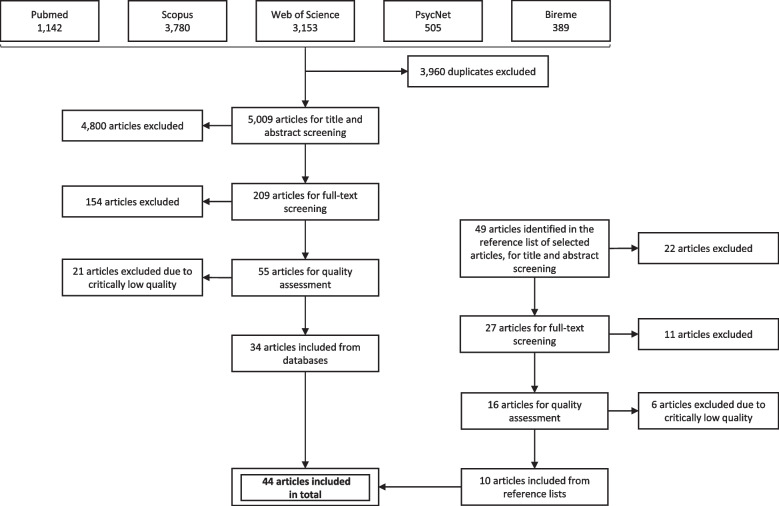
Flowchart of study screening and selection process

Study selection was carried out in two stages. First, we read titles and abstracts of all identified articles. Those that did not present enough information to decide for their exclusion went to the full-text reading stage. In both stages, each paper was screened by two independent reviewers for eligibility. In case of divergence between reviewers, a third reviewer was consulted. Systematic reviews found in the reference list of the selected articles underwent the same process. The study screening and selection process is shown in Fig. 1.

EndNote® X8 was used to manage, store, and organize references and remove duplicate studies. Rayyan QCRI® was adopted to manage the study selection process.

### Data extraction

From each review included we obtained: 1) metadata (author and year of publication); 2) methodological aspects (number of primary studies, study designs, and age groups included); 3) physical activity domains (leisure, travel, work or education, and household); 4) barriers and facilitators investigated; and 5) the direction of association between factors investigated and domain-specific physical activity behaviour (positive, negative, or no evidence of association). Data extraction was done using a pre-defined, standardised form by one reviewer, and double-checked independently by a second reviewer. In case of disagreement between reviewers, a consensus was sought between them.

### Harmonisation of barriers and facilitators and direction of association

The selected reviews reported barriers and facilitators in varied ways. Therefore, after data extraction, barriers and facilitators were grouped in common types by one reviewer, and then double-checked independently by other two reviewers. Disagreements were resolved through consensus. Table 2 lists the types of barriers and facilitators that are part of our review and examples of factors included in each case.

**Table 2:** Types and examples of barriers and facilitators investigated

Barriers and facilitators	Examples of factors investigated by the selected reviews
***Intrapersonal factors***	
Availability of personal equipment	Bicycle ownership, equipment for physical activity
Better skills	Motor skills, objective capacity to walk
Pleasure and fun with physical activity	Pleasure, enjoyment
Higher motivation and having goals	Intention, goal setting
Lack of time and presence of concurrent behaviours	Preference for sedentary behaviour, lack of time
Lower costs	Discretionary income, subscription fees
More positive beliefs about capabilities	Self-efficacy, perceived behaviour control
More positive beliefs about consequences	Attitude, perceived benefits
More/Better knowledge	Knowledge about exercise or health benefits
Negative emotions	Discomfort, pain
Positive past experiences	Past physical activity behaviour and experiences
Worse health condition	Self-rated health, nutritional status
***Social environment and interpersonal factors***	
Better/More positive general social support	Interpersonal influences, social support
Better/More positive social norms	Social practices, social norms
Better/More positive support from family	Support from parents or partners
Better/More positive support from friends	Peer support or pressure, time spent with friends
Better/More positive support from others	Support from school or health service staff
Higher physical activity of friends and family	Perceived physical activity habits of parents or friends
Worse perceived safety	Crime-related safety, parent’s safety concerns
***Built environment factors***	
Better general urban design and built environment	Residential or commercial density, city type
Better land use mix	Objective or perceived land-use mix
Better quality and condition of places	Aesthetics, maintenance
Better road safety	Traffic speed, safe pedestrian crossing
Better street connectivity	Objective or perceived street connectivity
Better public transport provision	Convenience or coverage of public transport
Better walkability	Objective or perceived walkability
Existence of active travel infrastructure	Availability of cycling or walking infrastructure
Existence of facilities within places	Play parks, amenities
Existence of, shorter distance to, and better access to places	Distance to destinations, perceived access to facilities
***Programmatic factors***	
Better quality of instructors	Instructor’s leadership or feedback quality
Better quality of physical activity programs	Number of activities, tailoring to participants’ skill level
Longer recess duration	More or longer school recesses
Participation in supervised activities	Involvement in structured activities

Intrapersonal factors were grouped based on domains suggested by Michie et al., [12] namely knowledge, skills, beliefs about capabilities, beliefs about consequences, motivation and goals, environmental context and resources, emotion, and nature of the behaviours. These domains are formed by a range of related constructs, so when intrapersonal factors identified by our review were associated only with a specific construct (e.g., availability of personal equipment) instead of the domain more generally (e.g., environmental context and resources), we decided to delimit the grouping at the construct level. Social environmental and interpersonal factors were grouped according to the source of support (family, friends, others, or general social support). We separated social norms from social support, as well as perceived support from role-modelling (physical activity behaviour of friends and family members), and dedicated a category to perceived safety. Grouping of built environment factors and programmatic factors was done a posteriori to best accommodate the barriers and facilitators as investigated by the reviews.

All types must have had at least two factors extracted from at least two separate review articles. Therefore, factors that did not fit within any of the types in Table 2 or could not be matched with factors from other reviews to create a new group were discarded (e.g., neuroticism, extroversion, physical activity intensity and frequency, time of the day, travel purpose (for travel physical activity), type of school recess, and use of physical activity monitors).

All barriers and facilitators in Table 2 were given a qualifier (e.g., better, worse), so that the direction of association with the outcome of interest (engagement in, or greater volume of, domain-specific physical activity) could be harmonised across reviews. For that, in the last stage before evidence synthesis, reviewers returned to their extractions and, when necessary, re-classified the direction of association according to the harmonised list of barriers and facilitators and outcome of interest.

### Evidence synthesis

For each pair of barrier/facilitator and domain-specific physical activity, we recorded the number of positive and negative associations reported across reviews, as well as when no evidence of association was observed.

### Quality assessment

Quality assessment of each systematic review was performed using the AMSTAR-2 tool [13]. Two reviewers independently classified the articles based on the 16 items of the instrument’s checklist. In case of disagreements, a meeting was held for discussion and consensus. AMSTAR-2 ranks the methodological quality of a systematic review as high, moderate, low, or critically low. Reviews classified as “critically low” were excluded from our review (Additional file 2, Table S1).

## Results

After screening and quality assessment, 44 systematic reviews [14–57] investigating the association between barriers and facilitators and domain-specific physical activity were included in our analysis. Table 3 presents study designs, age groups, domains of physical activity, and barriers and facilitators included by each systematic review, alongside the methodological quality rating. Thirty-three reviews included original studies with longitudinal designs (e.g., experiments, prospective cohorts). However, evidence from cross-sectional studies predominated in 26 out of 34 reviews that included this type of study design. Eleven reviews included qualitative studies and four considered mixed-methods approaches. Regarding the age groups investigated, 15 reviews included children, 22 included adolescents, 25 included adults, and 22 older adults. Leisure was the most investigated physical activity domain (*n* = 32), followed by travel (*n* = 22), work or education (*n* = 6), and household (*n* = 1).

**Table 3:** Studies included in the review

First author and publication year	Study designs	Population groups	Domains	Barriers and facilitators investigated	AMSTAR-2 rating
Abaraogu U 2018 [14]	8 cross-sectional 4 cohort 5 qualitative 1 mixed-methods	Older adults	Leisure	Better skills Better/More positive general social support Existence of facilities within places Higher motivation and having goals More positive beliefs about consequences More/Better knowledge Participation in supervised activities Worse health condition	Moderate
Aranda-Balboa M 2020 [15]	27 cross-sectional	Adults	Travel	Better general urban design and built environment Better road safety Better street connectivity Better/More positive general social support Existence of, shorter distance to, and better access to places Worse perceived safety	Moderate
Broekhuizen K 2014 [16]	17 observational 16 experimental	Children and adolescents	Education	Availability of personal equipment Better general urban design and built environment Existence of active travel infrastructure Existence of facilities within places Longer recess duration Participation in supervised activities Quality and condition of places Worse perceived safety	Moderate
Brunton G 2005 [17]	5 experimental 5 qualitative	Children and adolescents	Leisure	Better public transport provision Better road safety Better skills Better/More positive social norms Better/More positive support from family Better/More positive support from friends Existence of active travel infrastructure Existence of, shorter distance to, and better access to places Pleasure and fun with physical activity Higher motivation and having goals Higher physical activity of friends and family Lack of time and presence of concurrent behaviours Lower costs More positive beliefs about consequences Negative emotions Quality and condition of places Worse perceived safety	Moderate
Bunn F 2008 [18]	6 cross-sectional 4 experimental 14 qualitative	Adults	Leisure	Better quality of instructors Better quality of physical activity programs Better/More positive general social support More positive beliefs about capabilities More positive beliefs about consequences More/Better knowledge Negative emotions Positive past experiences	Moderate
Congello N 2018 [19]	1 experimental 3 qualitative 3 mixed-methods	Adults and older adults	Leisure	Better public transport provision Better/More positive social norms Better/More positive support from friends Lack of time and presence of concurrent behaviours Worse perceived safety	Moderate
Craike M 2019 [20]	67 cross-sectional 6 cohort	Adults	Leisure	Better walkability Better/More positive general social support Better/More positive social norms Better/More positive support from family Better/More positive support from friends More positive beliefs about capabilities More positive beliefs about consequences Quality and condition of places Worse health condition Worse perceived safety	Low
D’Haese S 2015 [21]	61 cross-sectional 4 cohort	Children	Leisure and travel	Better general urban design and built environment Better land use mix Better road safety Better street connectivity Better walkability Existence of active travel infrastructure Existence of facilities within places Existence of, shorter distance to, and better access to places Quality and condition of places Worse perceived safety	Low
Day K 2018 [22]	143 cross-sectional 16 cohort	Adolescents, adults, and older adults	Leisure, travel, and work/education	Better general urban design and built environment Better road safety	Low
Dennett R 2020 [23]	41 experimental	Adults and older adults	Leisure	Better/More positive general social support Better/More positive support from friends Better/More positive support from others Higher motivation and having goals More/Better knowledge	High
Elshahat S 2020 [24]	32 cross-sectional 1 case study	Adults and older adults	Leisure and travel	Better general urban design and built environment Better land use mix Better public transport provision Better road safety Better street connectivity Better walkability Existence of active travel infrastructure Existence of facilities within places Existence of, shorter distance to, and better access to places Quality and condition of places Worse perceived safety	Moderate
Escalante Y 2014 [25]	8 experimental	Children and adolescents	Work/Education	Existence of facilities within places Quality and condition of places	Low
Farrance C 2016 [26]	5 experimental 3 qualitative 2 mixed-methods	Older adults	Leisure	Better quality of instructors Better quality of physical activity programs Better/More positive social norms More positive beliefs about consequences Positive past experiences	Moderate
Hilland T 2020 [27]	32 cross-sectional 3 cohort	Adults and older adults	Leisure and travel	Better general urban design and built environment Better walkability Better/More positive general social support Better/More positive social norms Existence of, shorter distance to, and better access to places More positive beliefs about consequences Quality and condition of places Worse health condition Worse perceived safety	Moderate
Hutzler Y 2010 [28]	7 cross-sectional 12 experimental 4 qualitative	Adolescents and adults	Leisure	Better public transport provision Better skills Better/More positive general social support Better/More positive support from family More positive beliefs about capabilities More positive beliefs about consequences	Low
Ikeda E 2018 [29]	31 cross-sectional 5 cohort 1 case–control	Children and adolescents	Travel	Better land use mix Better road safety Better walkability Better/More positive social norms Better/More positive support from family Better/More positive support from friends Better/More positive support from others Existence of active travel infrastructure Existence of, shorter distance to, and better access to places Quality and condition of places Worse perceived safety	Moderate
Jaarsma E 2014 [30]	51 cross-sectional 3 cohort 3 experimental	Adolescents, adults, and older adults	Leisure	Better general urban design and built environment Better skills Better/More positive social norms Existence of, shorter distance to, and better access to places Pleasure and fun with physical activity Higher motivation and having goals Lack of time and presence of concurrent behaviours Lower costs More positive beliefs about consequences More/Better knowledge Worse health condition	Moderate
Kärmeniemi M 2018 [31]	21 cohort 30 experimental	Children, adolescents, adults, and older adults	Leisure and travel	Better general urban design and built environment Better walkability Existence of active travel infrastructure Existence of facilities within places Existence of, shorter distance to, and better access to places Quality and condition of places Worse perceived safety	Moderate
Liangruenrom M 2019 [32]	167 cross-sectional	Children, adolescents, adults, and older adults	Leisure, travel, work/education, and household	Better general urban design and built environment Better/More positive general social support Better/More positive support from family Better/More positive support from friends Better/More positive support from others Existence of, shorter distance to, and better access to places Pleasure and fun with physical activity Lack of time and presence of concurrent behaviours More positive beliefs about capabilities More positive beliefs about consequences More/Better knowledge Positive past experiences Worse health condition	Moderate
Lindsay Smith G 2017 [33]	22 cross-sectional 3 cohort 2 experimental	Older adults	Leisure	Better/More positive general social support Negative emotions	Moderate
Lorenc T 2008 [34]	16 qualitative	Children, adolescents, and adults	Travel	Better general urban design and built environment Better road safety Better skills Better/More positive general social support Better/More positive social norms Better/More positive support from family Better/More positive support from friends Better/More positive support from others Existence of active travel infrastructure Existence of, shorter distance to, and better access to places Pleasure and fun with physical activity Higher motivation and having goals Lack of time and presence of concurrent behaviours Lower costs More positive beliefs about capabilities More positive beliefs about consequences Negative emotions Quality and condition of places Worse health condition Worse perceived safety	Moderate
Maitland C 2013 [35]	38 observational 11 experimental	Adolescents	Leisure	Availability of personal equipment Better/More positive support from family Higher physical activity of friends and family	Moderate
Mendonça G 2014 [36]	64 cross-sectional 9 cohort 2 experimental	Adolescents	Leisure and travel	Better/More positive support from family Better/More positive support from friends	Low
Olekszechen N 2016 [37]	25 cross-sectional 3 experimental 5 qualitative	Adults	Travel	Availability of personal equipment Better land use mix Better/More positive general social support Better/More positive social norms Existence of facilities within places Lack of time and presence of concurrent behaviours More positive beliefs about consequences Negative emotions Positive past experiences	Low
Pan X 2021 [38]	14 cross-sectional 2 cohort 5 experimental	Children and adolescents	Travel	Existence of active travel infrastructure Quality and condition of places Worse perceived safety	High
Pollard T 2017 [39]	36 cross-sectional	Adults and older adults	Leisure and travel	Pleasure and fun with physical activity	High
Pont K 2009 [40]	38 cross-sectional	Children and adolescents	Travel	Better general urban design and built environment Better road safety Existence of active travel infrastructure Existence of, shorter distance to, and better access to places Worse perceived safety	Moderate
Rhodes R 2013 [41]	8 cross-sectional 52 cohort	Adolescents, adults, and older adults	Leisure	Existence of, shorter distance to, and better access to places Pleasure and fun with physical activity Higher motivation and having goals Lack of time and presence of concurrent behaviours More positive beliefs about capabilities Negative emotions Positive past experiences Quality and condition of places Worse perceived safety	Moderate
Rhodes R 2020 [42]	37 cross-sectional 9 cohort	Children, adolescents, adults, and older adults	Leisure and travel	Better/More positive general social support Better/More positive social norms Higher motivation and having goals More positive beliefs about consequences	Moderate
Ridgers N 2012 [43]	42 cross-sectional 11 not specified	Children and adolescents	Work/Education	Better quality of physical activity programs Better/More positive general social support Better/More positive social norms Existence of facilities within places Existence of, shorter distance to, and better access to places Pleasure and fun with physical activity Higher motivation and having goals Lack of time and presence of concurrent behaviours Longer recess duration Participation in supervised activities Quality and condition of places	Moderate
Rothman L 2018 [44]	61 cross-sectional 1 case–control 1 mixed-methods	Children and adolescents	Travel	Better general urban design and built environment Better road safety Existence of active travel infrastructure More positive beliefs about consequences Worse perceived safety	Moderate
Salvo G 2018 [45]	36 qualitative	Adults and older adults	Leisure and travel	Better road safety Better street connectivity Better/More positive social norms Existence of active travel infrastructure Existence of facilities within places Existence of, shorter distance to, and better access to places Negative emotions Quality and condition of places Worse perceived safety	Low
Scarapicchia T 2017 [46]	20 cohort	Adults	Leisure	Availability of personal equipment Better/More positive general social support Better/More positive support from family Better/More positive support from friends Positive past experiences	Moderate
Smith M 2017 [47]	15 cross-sectional 12 cohort 1 experimental	Children, adolescents, adults, and older adults	Travel	Better general urban design and built environment Better land use mix Better public transport provision Better road safety Better street connectivity Existence of active travel infrastructure Existence of facilities within places Existence of, shorter distance to, and better access to places Quality and condition of places	Moderate
Stanley R 2012 [48]	17 cross-sectional 5 experimental	Adolescents	Leisure and work/education	Availability of personal equipment Better land use mix Better quality of physical activity programs Better/More positive general social support Better/More positive support from family Better/More positive support from friends Better/More positive support from others Existence of active travel infrastructure Existence of facilities within places Existence of, shorter distance to, and better access to places Pleasure and fun with physical activity Higher physical activity of friends and family Longer recess duration More positive beliefs about capabilities More positive beliefs about consequences Participation in supervised activities Quality and condition of places Worse perceived safety	Moderate
Stappers N 2018 [49]	1 cross-sectional 4 cohort 10 experimental 4 not specified	Adults	Leisure and travel	Better general urban design and built environment Better public transport provision Existence of active travel infrastructure Existence of, shorter distance to, and better access to places	Low
Tovar M 2018 [50]	21 cross-sectional	Adults and older adults	Leisure	Better/More positive general social support More positive beliefs about capabilities Worse health condition Worse perceived safety	Moderate
Van Cauwenberg J 2011 [51]	28 cross-sectional 3 cohort	Older adults	Leisure and travel	Better general urban design and built environment Better land use mix Better public transport provision Better road safety Better street connectivity Better walkability Existence of active travel infrastructure Existence of, shorter distance to, and better access to places Quality and condition of places Worse perceived safety	Moderate
Van Cauwenberg J 2018 [52]	71 cross-sectional 1 cohort	Older adults	Leisure	Better land use mix Better public transport provision Existence of active travel infrastructure Existence of, shorter distance to, and better access to places Quality and condition of places	Low
Van Hecke L 2018 [53]	14 cross-sectional 17 qualitative	Adolescents	Leisure	Availability of personal equipment Better public transport provision Better road safety Existence of active travel infrastructure Existence of facilities within places Quality and condition of places	Moderate
Van Holle V 2012 [54]	69 cross-sectional 1 cohort	Adults and older adults	Leisure and travel	Better general urban design and built environment Better public transport provision Better road safety Better walkability Existence of active travel infrastructure Existence of, shorter distance to, and better access to places Quality and condition of places Worse perceived safety	Moderate
Xiao C 2019 [55]	9 experimental	Adults	Travel	Better public transport provision	Moderate
Yarmohammadi, S 2019 [56]	20 cross-sectional 14 qualitative	Older adults	Leisure	Better general urban design and built environment Better/More positive general social support Existence of facilities within places Higher motivation and having goals Lack of time and presence of concurrent behaviours Lower costs More positive beliefs about consequences Negative emotions Participation in supervised activities Quality and condition of places Worse health condition	Moderate
Zhang R 2019 [57]	25 cross-sectional	Children, adolescents, adults, and older adults	Leisure	Better road safety Better/More positive social norms Better/More positive support from family Better/More positive support from friends Existence of active travel infrastructure Existence of facilities within places Existence of, shorter distance to, and better access to places Quality and condition of places Worse perceived safety	Moderate

Factors related to the built environment were the most investigated among the reviews: 22 investigated the existence of, distance to, and/or access to spaces; 24 explored the quality and condition of these spaces; and 20 reviews investigated active travel infrastructure. The social environment and interpersonal factors came after, with 22 reviews looking at perceived safety and 19 at general social support. The most investigated intrapersonal factor was beliefs about physical activity consequences (*n* = 16).

### Quality assessment

The overall confidence in the results of 27 reviews was rated as critically low (Additional file 2, Table S1), which were excluded from our analysis. Of the 44 reviews selected, 10 were classified as low quality, 31 as moderate quality, and three as high quality (Table 3 and Additional file 2, Table S2). Scores for each of the AMSTAR-2 16 items can be found in Additional file 2.

### Barriers and facilitators for leisure-time physical activity

Twenty-one reviews investigated intrapersonal barriers and facilitators for leisure-time physical activity [14, 17–20, 23, 26, 28, 30, 32, 33, 35, 39, 41, 42, 45, 46, 48, 50, 53, 56]. Better skills (5/5 synthesis units), higher motivation and goal setting (11/12), and positive beliefs about the physical activity consequences (18/21) were consistently associated with higher levels of leisure-time physical activity. Experiencing pleasure and fun with physical activity (6/8) and more/better knowledge about physical activity (6/8) were also associated with higher levels of practice, albeit the evidence was less consistent.

On the other hand, lack of time and easy access to concurrent behaviours (8/9), negative emotions related to physical activity practice (7/8), and worse health conditions (7/9) were negatively associated with leisure-time physical activity levels (Table 4).

**Table 4:** Number of synthesis units showing negative (-), positive ( +), and no evidence (o) of association observed between barriers and facilitators and higher levels of domain-specific physical activity

**Barriers and facilitators**	**Leisure**			**Travel**			**Work or education**		
	**-**	**o**	** + **	**-**	**o**	** + **	**-**	**o**	** + **
***Intrapersonal factors***									
Availability of personal equipment	0	4	1	0	0	1	0	2	0
Better skills	0	0	5	0	2	0	0	0	0
Pleasure and fun with physical activity	0	2	6	0	1	0	0	1	0
Higher motivation and having goals	0	1	11	0	2	0	0	0	1
Lack of time and presence of concurrent behaviours	8	1	0	2	1	0	1	0	0
Lower costs	1	0	2	0	1	0	0	0	0
More positive beliefs about capabilities	0	6	7	0	2	0	0	0	0
More positive beliefs about consequences	0	3	18	0	2	7	0	0	0
More/Better knowledge	0	2	6	0	0	0	0	0	0
Negative emotions	7	1	0	3	2	0	0	0	0
Positive past experiences	0	4	3	0	0	1	0	0	0
Worse health condition	7	2	0	0	1	0	1	0	0
***Social environment and interpersonal factors***									
Better/More positive general social support	0	5	12	0	3	2	0	0	1
Better/More positive social norms	0	4	5	0	7	5	1	0	0
Better/More positive support from family	0	6	11	0	4	0	0	0	0
Better/More positive support from friends	0	4	6	0	2	1	0	0	0
Better/More positive support from others	0	2	1	0	3	0	0	1	0
Higher physical activity of friends and family	0	3	1	0	0	0	0	0	0
Worse perceived safety	6	14	0	12	12	0	0	2	0
***Built environment factors***									
Better general urban design and built environment	0	8	5	1	10	12	0	2	2
Better land use mix	0	5	1	0	4	5	0	0	0
Better quality and condition of places	0	15	11	0	9	6	0	12	8
Better road safety	0	8	5	0	14	9	0	0	0
Better street connectivity	0	4	2	0	6	5	0	0	0
Better public transport provision	0	2	6	0	6	4	0	0	0
Better walkability	0	8	2	0	1	7	0	0	0
Existence of active travel infrastructure	0	13	7	0	17	12	0	0	1
Existence of facilities within places	0	24	11	0	5	11	8	55	12
Existence of, shorter distance to, and better access to places	0	20	11	0	17	17	0	0	1
***Programmatic factors***									
Better quality of instructors	0	1	1	0	0	0	0	0	0
Better quality of physical activity programs	0	3	1	0	0	0	0	1	1
Longer recess duration	0	0	0	0	0	0	0	3	2
Participation in supervised activities	0	1	3	0	0	0	2	6	0

Twenty-three reviews investigated social environment and interpersonal factors [14, 17, 19–21, 23, 24, 26–28, 30–32, 35, 36, 45, 46, 48, 50, 51, 54, 56, 57]. Evidence of moderate consistency indicates that general social support (12/17) and support from family members (11/17) are positively associated with leisure-time physical activity practice. No evidence of association between perceived safety and leisure-time physical activity was observed in 14 out of 20 synthesis units (Table 4).

Twenty-two reviews investigated built environment barriers and facilitators [14, 17, 19–22, 24, 27, 28, 30–32, 41, 45, 48, 49, 51–54, 56, 57]. No consistent evidence of association was observed, except for some evidence indicating that public transport provision might be a facilitator (6/8). No evidence of association was observed in approximately two thirds or more of the synthesis units involving the existence of active travel infrastructure (13/20); existence, distance, and access to places for physical activity (20/31); existence of facilities within these places (24/35); land use mix (5/6); and walkability (8/10) (Table 4).

Evidence on programmatic factors was limited [18, 26, 48] and no consistent association was observed with leisure-time physical activity (Table 4).

### Barriers and facilitators for travel-related physical activity

Six reviews investigated [32, 34, 37, 39, 44, 45] the association between intrapersonal factors and levels of travel-related physical. Some evidence indicating that beliefs about the physical activity consequences (7/9) are positively associated with active travel (Table 4).

Evidence from 17 reviews [15, 21, 24, 27, 29, 31, 34, 36–38, 40, 42, 44, 45, 51, 54] was mixed for social environment and interpersonal factors, particularly for general social support (2/5), social norms (5/12), and perceived safety (12/24), with no consistent association observed. No evidence of association was observed in all four synthesis units between support from family members and travel-related physical activity (Table 4).

As for built environment factors, evidence from 19 reviews [15, 21, 22, 24, 27, 29, 31, 32, 34, 37, 38, 40, 44, 45, 47, 49, 51, 54, 55] showed moderate consistency of positive association between travel-related physical activity and walkability (7/8) and existence of facilities that support active travel (11/16). Evidence was mixed for the other factors (Table 4).

### Barriers and facilitators for physical activity at work, educational and domestic settings

Evidence on barriers and facilitators for physical activity at the work, educational and domestic settings is very scarce and, overall, showed limited consistency. Regarding physical activity at work and educational settings [16, 22, 25, 32, 43, 48], the most explored factors are quality and condition of places for physical activity and existence of facilities within these places, with most of the synthesis units indicating no evidence of association (12/20 and 55/75, respectively) (Table 4). Evidence of low consistency showed that household physical activity levels were negatively associated with health conditions (one synthesis unit) and positively associated with general urban design and built environment (two synthesis units) [32].

## Discussion

Our systematic review of reviews provides the most comprehensive overview up to this date of the current evidence base on barriers and facilitators of domain-specific physical activity behaviour. Our findings show that the evidence base is largest for leisure-time, followed by travel-related physical activity, whereas a very limited number of reviews were dedicated to physical activity in work, educational and domestic settings. Across all physical activity domains, factors related to the built environment were more abundant in the reviews than intra and interpersonal factors, and almost no reviews investigated programmatic factors. Very consistent associations could be observed between a range of intrapersonal factors and leisure-time physical activity. Almost no reviews synthesized the association between intrapersonal factors and physical activity in the other domains. Results for social and built environmental factors were moderately consistent at the best, across all domains.

Our study has some limitations. For some barriers or facilitators, the most recent systematic review might have been conducted years ago, so our findings might not incorporate the results of the most recent original studies. Since the COVID-19 pandemic, a number of studies have been conducted to investigate changes in physical activity behaviour that occurred in this new scenario, and the factors associated with these behavioural changes. However, a rapid search and screening conducted in September 2022 found only two additional systematic reviews (out of 5153 entries) published since September 2020 that focused on barriers and facilitators of domain-specific physical activity behaviour [58, 59], whose results were largely in line with the findings of this study. Second, we used a very broad inclusion criteria to capture as much of the evidence available as possible. Consequently, the reviews included in our study vary in the inclusion criteria they applied, for instance, in terms of population groups (e.g., only men or women, specific age groups, only socially or economic disadvantaged people), locations (e.g., urban or rural, specific regions of the globe), and methodological design of the original studies. It is possible that certain barriers and facilitators are more consistently associated to domain-specific physical activity in some groups of the population or locations than others, and that combining the results of all reviews might mask these patterns. Third, the quality and interpretation of our synthesis are affected by the methodological quality of the reviews, and the original studies they included. Of the 44 reviews included in our study, only three were rated as having high methodological quality according to AMSTAR-2, with other 31 rated as having moderate quality. Moreover, we excluded 27 reviews from our synthesis because their critically low methodological quality according to AMSTAR-2. Of the 44 reviews included, thirty-four included cross-sectional studies, which was the predominant study design in 26 of these reviews. Fourth, the reviews and the original studies they included might differ in their operational definition of a same barrier or facilitator, and in how they measured domain-specific physical activity.

As expected, the evidence base is larger for the leisure domain, in all groups of factors investigated. We observed consistent evidence of association for a range of intrapersonal factors, including better skills, higher motivation and goal setting, positive beliefs about the physical activity consequences, lack of time and easy access to concurrent behaviours, negative emotions related to physical activity practice, and worse health conditions. We also found evidence of moderate consistency that general social support and support from family members are positively associated with leisure-time physical activity. Results were largely mixed for built environment factors, with results indicating either positive or no evidence of association. Because of the volitional nature of leisure activities, personal predisposition is a necessary condition to engage in recreational physical activity. Even though social and built environment factors can contribute to behavioural adoption and maintenance, their effects might not be observed if personal predisposition to engage in physical activity does not reach a certain threshold, which can explain the more mixed results observed for these two sets of factors.

Attention to the factors that enable and prevent travel-related physical activity has been increasing, but very much related to the built environment. Evidence of moderate consistency indicated a positive relationship with walkability and existence of facilities that support active travel. However, travel behaviour, including active travel, is shaped by influences at the macro, meso, and micro level [60]. The disproportional emphasis in the macro level (built environment) with limited understanding of the micro- and meso-level factors that affect travel decisions and behaviours very likely will be insufficient to design effective active travel promotion strategies. For instance, travel decisions are affected by circumstantial factors (e.g., journey purpose) that make one mode (e.g., bicycle) more or less appealing for a given journey. Also, Mattioli et al. suggest that some people might be consciously dependent of a mode (e.g., car) regardless of other circumstances (e.g., availability of other modes) [60]. Therefore, more work is needed to understand what factors, at all levels, affect active travel behaviour.

The evidence gap is even more salient for facilitators and barriers of physical activity in work, educational and domestic settings. Even though different domains of physical activity may impact health in different ways [6, 61, 62] public health messaging encourages that physical activity be incorporated throughout the day (i.e., in different domains) as part of an active lifestyle [1]. However, reductions in work and domestic physical activity have been observed and forecasted across the globe as a result of economic and social transitions [63], with likely larger impacts in low- and middle-income countries, where these domains respond for the larger fraction of physical activity volume in adults [5]. Hence, understanding what facilitates and prevents physical activity in work, educational and domestic settings is as important as in the leisure and transport domains for a successful day-long approach to physical activity promotion.

It is important to acknowledge that most of the original studies in the reviews we included were conducted in high-income Western settings, and that what facilitates or prevents domain-specific physical activity might be different in other locations, due to cultural, socio-economic, and environmental differences, for instance. Also, even though the evidence base was summarized for each factor in isolation, these factors are likely interdependent, with different combinations of factors affecting differently the capability, opportunity, and motivation [64] to be more active within and across physical activity domains. Hence, it is important that future studies on facilitators and barriers consider the broader context and underlying conditions in which specific factors seem to be more or less likely to affect physical activity behaviour.

Looking at the findings by categories of barriers and facilitators, built environment factors accounted for 447 synthesis units, equivalent to 60% of all units investigated in this study. This is more than three times the number of synthesis units in the intrapersonal (*n* = 142) and social environment and interpersonal (*n* = 136) categories. Only 25 synthesis units have been reported about programmatic factors. This imbalance in the evidence base is even more evident when we consider the domains. For instance, almost 70% of all synthesis units in the travel domain are related to built environment, more than six times the number of units for intrapersonal factors (11%). Even though there is a consensus in the field that physical activity is a multi-dimensional, multi-factorial behaviour, it is evident that there has been a disproportional emphasis in the investigation of built environment barriers and facilitators that needs to be addressed in the future.

Our study indicates a number of research opportunities, across both domains and categories of barriers and facilitators, that need to be addressed if we want to have a better understanding of the factors that affect domain-specific physical activity behaviour. First, there is a clear deficit of evidence of barriers and facilitators in work and domestic settings, which correspond to the larger portion of the moderate-to-vigorous physical activity volume [5]. Second, it seems that most of the evidence generated so far is about built environment factors, even though evidence still uncertain about a number of potential intrapersonal and interpersonal barriers and facilitators. Third, it is important to acknowledge that these barriers and facilitators, and perhaps the domains, very likely interact between themselves over the day, with different combinations of factors creating sufficient conditions to have more physically (in)active lifestyle patterns. These knowledge gaps limit a holistic understanding of the conditions that affect domain-specific physical activity behaviour and, consequently, the design of promotion strategies that can effectively incentivize and sustain more active lifestyles.

## Conclusion

Our study provides a picture of the research conducted so far on intrapersonal, interpersonal, and built environment factors that can prevent or facilitate physical activity behaviour across domains. Even though it is accepted that knowledge of the barriers and facilitators of physical activity across domains is necessary to design promotion strategies that support more active lifestyles over the day, the evidence base is still limited and biased towards the leisure domain and built environment factors. Efforts and resources are required to diversify and strength the evidence base required to create the conditions for more physically active societies.

## Supplementary Information

**Additional file 1. **Search string for Scopus. Search string for PsycNET. Search string for PubMed. Search string for Bireme. Search string for ISI Web of Science.

**Additional file 2: Table S1.** AMSTAR-2 assessment of systematic reviews with critically low quality.** Table S2.** AMSTAR-2 assessment of selected systematic reviews.

## Data Availability

The datasets used and analysed during the current study are available from the corresponding author on reasonable request.

## Abbreviations

PRISMA: Preferred Reporting Items for Systematic Reviews and Meta-analysis guidelines
PROSPERO: International Prospective Register of Systematic Reviews
BIREME: Regional Library of Medicine
