# Supplementary material for: Barriers and facilitators of domain-specific physical activity: a systematic review of reviews
Source: BMC Public Health. 2022 Oct 26;22:1964. doi: 10.1186/s12889-022-14385-1 (PMC9598005; doi:10.1186/s12889-022-14385-1)
Supplement: Supplementary file 1 — Additional file 1. Search string for Scopus. Search string for PsycNET. Search string for PubMed. Search string for Bireme. Search string for ISI Web of Science. [file 12889_2022_14385_MOESM1_ESM.docx]

**Search string for Scopus**

| #1: Physical activity | TITLE-ABS-KEY("motor activity") OR TITLE-ABS-KEY(exercise) OR TITLE-ABS-KEY("Physical Education") OR TITLE-ABS-KEY("physical activity") OR TITLE-ABS-KEY("recreational activity") OR TITLE-ABS-KEY(sport) OR TITLE-ABS-KEY(sports) OR TITLE-ABS-KEY(sedentary) OR TITLE-ABS-KEY("physical inactivity") OR TITLE-ABS-KEY("active transport") OR TITLE-ABS-KEY("active transportation") OR TITLE-ABS-KEY("active commut*") OR TITLE-ABS-KEY("active travel") OR TITLE-ABS-KEY(bicycle) OR TITLE-ABS-KEY(bicycling) OR TITLE-ABS-KEY(bike) OR TITLE-ABS-KEY(biking) OR TITLE-ABS-KEY(walk) OR TITLE-ABS-KEY(walking) OR TITLE-ABS-KEY("leisure activities") OR TITLE-ABS-KEY(dancing) OR TITLE-ABS-KEY(gardening) OR TITLE-ABS-KEY("activities of daily living") |
| --- | --- |
| #2: Barriers and facilitators | TITLE-ABS-KEY(covariates) OR TITLE-ABS-KEY(correlates) OR TITLE-ABS-KEY(determinants) OR TITLE-ABS-KEY(mediators) OR TITLE-ABS-KEY(moderators) OR TITLE-ABS-KEY(predictors) OR TITLE-ABS-KEY(environment) OR TITLE-ABS-KEY(contributors) OR TITLE-ABS-KEY(facilitators) OR TITLE-ABS-KEY(barriers) |
| #3: Study type | TITLE-ABS-KEY("systematic review") OR TITLE-ABS-KEY(meta-analysis) OR TITLE-ABS-KEY("umbrella review") OR TITLE-ABS-KEY("review of reviews") |
| #4 | #1 AND #2 AND #3  *Filters:* Language = English, Spanish, Portuguese. |

**Search string for PsycNET**

| #1: Physical activity | MeSH: motor activity |
| --- | --- |
| #2: Physical activity | Keywords: (exercise OR "Physical Education” OR "physical activity” OR "recreational activity” OR sport OR sports OR sedentary OR "physical inactivity” OR "active transport” OR "active transportation” OR "active commut*” OR "active travel” OR bicycle OR bicycling OR bike OR biking OR walk OR walking OR "leisure activities” OR dancing OR gardening OR "activities of daily living”) |
| #3: Physical activity | Abstract: (exercise OR "Physical Education” OR "physical activity” OR "recreational activity” OR sport OR sports OR sedentary OR "physical inactivity” OR "active transport” OR "active transportation” OR "active commut*” OR "active travel” OR bicycle OR bicycling OR bike OR biking OR walk OR walking OR "leisure activities” OR dancing OR gardening OR "activities of daily living”) |
| #4: Physical activity | #1 OR #2 OR #3 |
| #5: Barriers and facilitators | Keywords: (covariates OR correlates OR determinants OR mediators OR moderators OR predictors OR environment OR contributors OR facilitators OR barriers) |
| #6: Barriers and facilitators | Abstract: (covariates OR correlates OR determinants OR mediators OR moderators OR predictors OR environment OR contributors OR facilitators OR barriers) |
| #7: Barriers and facilitators | #5 OR #6 |
| #8: Study type | Keywords: (“systematic review” OR meta-analysis OR “umbrella review” OR “review of reviews”) |
| #9: Study type | Abstract: (“systematic review” OR meta-analysis OR “umbrella review” OR “review of reviews”) |
| #10: Study type | #8 OR #9 |
| #11: Languages | Language: (English OR Portuguese OR Spanish) |
| #12 | #4 AND #7 AND #10 AND #11  *Filters:* Population group = Human. |

**Search string for PubMed**

| #1: Physical activity | ("motor activity" [MeSH Terms]) OR (exercise [MeSH Terms]) OR ("Physical Education" [Title/Abstract]) OR ("physical activity" [Title/Abstract]) OR ("recreational activity" [Title/Abstract]) OR (sport* [Title/Abstract]) OR (sedentary [Title/Abstract]) OR ("physical inactivity" [Title/Abstract]) OR ("active transport" [Title/Abstract]) OR ("active transportation" [Title/Abstract]) OR ("active commut*" [Title/Abstract]) OR ("active travel*" [Title/Abstract]) OR (bicycle [Title/Abstract]) OR (bicycling [MeSH Terms]) OR (bike [Title/Abstract]) OR (biking [Title/Abstract]) OR (walk [Title/Abstract]) OR (walking [MeSH Terms]) OR ("leisure activities" [MeSH Terms]) OR (dancing [MeSH Terms]) OR (gardening [MeSH Terms]) OR ("activities of daily living" [MeSH Terms]) |
| --- | --- |
| #2: Barriers and facilitators | (covariates [Title/Abstract]) OR (correlates [Title/Abstract]) OR (determinants [Title/Abstract]) OR (mediators [Title/Abstract]) OR (moderators [Title/Abstract]) OR (predictors [Title/Abstract]) OR (environment [MeSH Terms]) OR (contributors [Title/Abstract]) OR (facilitators [Title/Abstract]) OR (barriers [Title/Abstract]) |
| #3: Study type | (“systematic review” [Title/Abstract]) OR (“meta-analysis” [Title/Abstract]) OR ("umbrella review" [Title/Abstract]) OR ("review of reviews" [Title/Abstract]) |
| #4: Humans | (Humans [MeSH Terms]) |
| #5 | #1 AND #2 AND #3 AND #4  *Filters:* Humans, English, Portuguese, Spanish |

**Search string for Bireme**

| #1: Physical activity | (tw:("motor activity")) OR (tw:(exercise)) OR (tw:("physical education")) OR (tw:("physical activity")) OR (tw:("recreational activity")) OR (tw:(sport)) OR (tw:(sports)) OR (tw:(sedentary)) OR (tw:("physical inactivity")) OR (tw:("active travel")) OR (tw:(bicycle)) OR (tw:(bicycling)) OR (tw:(bike)) OR (tw:(biking)) OR (tw:(walk)) OR (tw:(walking)) OR (tw:("leisure activities")) OR (tw:(dancing)) OR (tw:(gardening)) OR (tw:("activities of daily living")) |
| --- | --- |
| #2: Barriers and facilitators | (tw:(covariates)) OR (tw:(correlates)) OR (tw:(determinants)) OR (tw:(mediators)) OR (tw:(moderators)) OR (tw:(predictors)) OR (tw:(environment)) OR (tw:(contributors)) OR (tw:(facilitators)) OR (tw:(barriers)) |
| #3: Study type | (tw:("systematic review")) OR (tw:("meta-analysis")) OR (tw:("umbrella review")) OR (tw:("review of reviews")) |
| #4: | #1 AND #2 AND #3  *Filters:* Humans, English, Portuguese, Spanish |

**Search string for ISI Web of Science**

| #1: Physical activity | TS=("motor activity" OR exercise OR "Physical Education" OR "physical activity" OR "recreational activity" OR sport OR sedentary OR "physical inactivity" OR "active transport" OR "active transportation" OR "active commut*" OR "active travel" OR bicycle OR bicycling OR bike OR biking OR walk OR walking OR "leisure activities" OR dancing OR gardening OR "activities of daily living") |
| --- | --- |
| #2: Barriers and facilitators | TS=(covariates OR correlates OR determinants OR mediators OR moderators OR predictors OR environment OR contributors OR facilitators OR barriers) |
| #3 Study type | TS=("systematic review" OR "meta-analysis" OR "umbrella review" OR "review of reviews") |
| #4 | #1 AND #2 AND #3  *Filters:* English, Portuguese, Spanish |
