# Supplementary material for: Barriers and facilitators of domain-specific physical activity: a systematic review of reviews
Source: BMC Public Health. 2022 Oct 26;22:1964. doi: 10.1186/s12889-022-14385-1 (PMC9598005; doi:10.1186/s12889-022-14385-1)
Supplement: Supplementary file 2 — Additional file 2: Table S1. AMSTAR-2 assessment of systematic reviews with critically low quality. Table S2. AMSTAR-2 assessment of selected systematic reviews. [file 12889_2022_14385_MOESM2_ESM.docx]

| **Table S1.** AMSTAR-2 assessment of systematic reviews with critically low quality. | | | | | | | | | | | | | | | | | |
| --- | --- | --- | --- | --- | --- | --- | --- | --- | --- | --- | --- | --- | --- | --- | --- | --- | --- |
| **Study** | **1** | **2** | **3** | **4** | **5** | **6** | **7** | **8** | **9** | **10** | **11** | **12** | **13** | **14** | **15** | **16** | **Overall rating** |
| Arango 2013 | No | Partial Yes | No | Partial Yes | No | No | Yes | No | No | No | No MA | No MA | No | No | No | Yes | Critically low |
| Browall 2018 | No | No | No | Partial Yes | Yes | Yes | Partial Yes | Partial Yes | No | No | No MA | No MA | No | No | No | Yes | Critically low |
| Bult 2011 | No | Partial Yes | Yes | Partial Yes | Yes | Yes | Partial Yes | No | No | No | No MA | No MA | No | No | No | No | Critically low |
| Davison 2006 | Yes | No | Yes | No | No | No | No | Partial Yes | No | No | No MA | No MA | No | No | No | No | Critically low |
| Day 2016 | Yes | Partial Yes | Yes | Partial Yes | No | No | Partial Yes | Partial Yes | No | Yes | No MA | No MA | No | No | No | Yes | Critically low |
| Fraser 2010 | Yes | Partial Yes | Yes | Partial Yes | Yes | Yes | Yes | Partial Yes | No | Yes | No MA | No MA | No | Yes | No | Yes | Critically low |
| Hadgraft 2018 | Yes | Partial Yes | No | Partial Yes | Yes | Yes | Yes | No | No | No | No MA | No MA | No | No | No | No | Critically low |
| Humpel 2002 | No | Partial Yes | No | Partial Yes | No | No | Yes | No | No | No | No MA | No MA | No | No | No | No | Critically low |
| Johnson 2013 | No | No | No | No | Yes | No | No | No | No | No | No MA | No MA | No | No | No | Yes | Critically low |
| Kebbe 2017 | No | Partial Yes | No | Partial Yes | Yes | Yes | Yes | No | No | No | No MA | No MA | No | No | No | Yes | Critically low |
| Kramer 2017 | No | No | No | No | Yes | No | No | No | No | Yes | No | No | No | Yes | No | No | Critically low |
| Kuijsters-Timmers 2019 | Yes | Partial Yes | No | Partial Yes | Yes | Yes | Yes | No | No | No | No MA | No MA | No | No | No | No | Critically low |
| Lacy-Vawdon 2018 | No | Partial Yes | No | Partial Yes | Yes | Yes | No | Partial Yes | No | No | No MA | No MA | No | Yes | No | Yes | Critically low |
| Larouche 2015 | No | No | No | No | No | No | No | No | No | No | No MA | No MA | No | No | No | No | Critically low |
| Lee 2008 | No | No | No | Partial Yes | No | No | No | No | No | No | No MA | No MA | Yes | Yes | No | No | Critically low |
| Mayne 2015 | Yes | Partial Yes | Yes | No | No | Yes | Yes | Partial Yes | No | Yes | No MA | No MA | No | No | No | Yes | Critically low |
| Molenberg 2019 | Yes | Partial Yes | Yes | Partial Yes | Yes | Yes | Yes | No | No | No | No MA | No MA | No | No | No | Yes | Critically low |
| Moran 2014 | Yes | Partial Yes | Yes | Partial Yes | Yes | Yes | Partial Yes | Partial Yes | No | No | No MA | No MA | No | Yes | No | Yes | Critically low |
| NordbØ 2019 | Yes | Yes | No | Partial Yes | Yes | Yes | Yes | Partial Yes | No | No | No MA | No MA | Yes | Yes | No | No | Critically low |
| Oliveira 2014 | Yes | Partial Yes | Yes | Partial Yes | Yes | Yes | Partial Yes | Partial Yes | No | No | No MA | No MA | No | No | No | No | Critically low |
| Qiu 2017 | Yes | No | No | Partial Yes | Yes | Yes | No | No | No | No | No MA | No MA | No | No | No | Yes | Critically low |
| Rech 2018 | Yes | No | No | Partial Yes | Yes | Yes | Yes | No | No | No | No MA | No MA | No | No | No | Yes | Critically low |
| Rissel 2012 | Yes | Partial Yes | No | Partial Yes | Yes | Yes | No | Partial Yes | No | No | No MA | No MA | No | No | No | Yes | Critically Low |
| Rothman 2012 | Yes | No | No | Partial Yes | Yes | Yes | Yes | No | No | No | No MA | No MA | No | No | No | Yes | Critically low |
| Scheepers 2014 | No | Partial Yes | Yes | Partial Yes | Yes | No Partial Yes | No | No | No | Yes | No MA | No MA | No | Yes | No | Yes | Critically low |
| Sugiyama 2011 | Yes | No | No | Partial Yes | Yes | Yes | No | No | No | No | No MA | No MA | No | No | No | Yes | Critically low |
| Zapata-Diomedi 2016 | Yes | Yes | No | Partial Yes | No | No | Yes | No | No | No | No MA | No MA | Yes | Yes | No | Yes | Critically low |

Items: (1) Did the research questions and inclusion criteria for the review include the components of PICO?; (2) Did the report of the review contain an explicit statement that the review methods were established prior to the conduct of the review and did the report justify any significant deviations from the protocol?; (3) Did the review authors explain their selection of the study designs for inclusion in the review?; (4) Did the review authors use a comprehensive literature search strategy?; (5) Did the review authors perform study selection in duplicate?; (6) Did the review authors perform data extraction in duplicate?; (7) Did the review authors provide a list of excluded studies and justify the exclusions?; (8) Did the review authors describe the included studies in adequate detail?; (9) Did the review authors use a satisfactory technique for assessing the RoB in individual studies that were included in the review?; (10) Did the review authors report on the sources of funding for the studies included in the review?; (11) If MAs was performed did the review authors use appropriate methods for statistical combination of results?; (12) If MAs was performed, did the review authors assess the potential impact of RoB in individual studies on the results of the MAs or other evidence synthesis?; (13) Did the review authors account for RoB in individual studies when interpreting/ discussing the results of the review?; (14) Did the review authors provide a satisfactory explanation for, and discussion of, any heterogeneity observed in the results of the review?; (15) If they performed quantitative synthesis did the review authors carry out an adequate investigation of publication bias and discuss its likely impact on the results of the review?; (16) Did the review authors report any potential sources of conflict of interest, including any funding they received for conducting the review?; AMSTAR, Assessing the Methodological Quality of Systematic Reviews; MA, meta-analysis.

| **Table S2.** AMSTAR-2 assessment of selected systematic reviews. | | | | | | | | | | | | | | | | | |
| --- | --- | --- | --- | --- | --- | --- | --- | --- | --- | --- | --- | --- | --- | --- | --- | --- | --- |
| **Study** | **1** | **2** | **3** | **4** | **5** | **6** | **7** | **8** | **9** | **10** | **11** | **12** | **13** | **14** | **15** | **16** | **Overall rating** |
| Abaraogu 2018 | No | Partial Yes | No | Partial Yes | Yes | Yes | Yes | Partial Yes | No AM | Yes | No MA | No MA | Yes | Yes | No MA | Yes | Moderate |
| Aranda-Balboa 2019 | Yes | Partial Yes | No | Yes | Yes | Yes | No | Partial Yes | No AM | No | No MA | No MA | Yes | Yes | Yes | Yes | Moderate |
| Broekhuizen 2014 | Yes | Yes | Yes | Partial Yes | Yes | Yes | Yes | Partial Yes | Yes | No | No MA | No MA | Yes | Yes | No | Yes | Moderate |
| Brunton 2005 | Yes | Partial Yes | Yes | Partial Yes | Yes | Yes | Partial Yes | Partial Yes | Partial Yes | Yes | No MA | No AM | Yes | Yes | No | Yes | Moderate |
| Bunn 2008 | Yes | Partial Yes | Yes | Partial Yes | Yes | Yes | Partial Yes | Partial Yes | Partial Yes | Yes | No MA | No MA | Yes | Yes | No | Yes | Moderate |
| Congello 2018 | Yes | Partial Yes | Yes | Partial Yes | Yes | Yes | Partial Yes | No | Partial Yes | No | No MA | No MA | Yes | Yes | No | No | Moderate |
| Craike 2019 | Yes | Partial Yes | Yes | Partial Yes | Yes | Yes | Yes | Partial Yes | Partial Yes | Yes | No MA | No MA | No | No | No | Yes | Low |
| D’Haese 2015 | Yes | Yes | Yes | Partial Yes | Yes | Yes | Yes | No | No | No | No MA | No MA | Yes | Yes | No | Yes | Low |
| Day 2018 | Yes | Partial Yes | Yes | Partial Yes | Yes | Yes | Partial Yes | Partial Yes | No | Yes | No MA | No MA | Yes | Yes | No | Yes | Low |
| Dennett 2020 | Yes | Yes | Yes | Yes | Yes | Yes | Yes | Yes | Yes | Yes | Yes | Yes | Yes | Yes | Yes | Yes | High |
| Elshahat 2020 | Yes | Partial Yes | Yes | Partial Yes | Yes | Yes | Yes | Partial Yes | No | Partial Yes | No MA | No MA | Yes | Yes | No | Yes | Moderate |
| Escalante 2014 | Yes | Partial Yes | Yes | Partial Yes | No | No | Yes | Yes | Partial Yes | No | No MA | No MA | No | No | No | Yes | Low |
| Farrance 2016 | Yes | Partial Yes | Yes | Partial Yes | Yes | Yes | Yes | Partial Yes | No | Yes | No MA | No MA | Yes | Yes | No | Yes | Moderate |
| Hilland 2020 | Yes | Partial Yes | Yes | Partial Yes | Yes | Yes | Yes | Partial Yes | Partial Yes | Yes | No MA | No MA | Yes | Yes | No | Yes | Moderate |
| Hutzler 2010 | Yes | Partial Yes | Yes | Partial Yes | Yes | Yes | Yes | Partial Yes | Partial Yes | Yes | No MA | No MA | No | Yes | No | Yes | Low |
| Ikeda 2018 | Yes | Partial Yes | Yes | Partial Yes | Yes | Yes | Yes | Partial Yes | Partial Yes | Yes | No | No | Yes | Yes | No | Yes | Moderate |
| Jaarsma 2014 | Yes | Partial Yes | Yes | Partial Yes | Yes | Yes | Yes | No | Partial Yes | No | No MA | No MA | Yes | No | No | No | Moderate |
| Karmeniemi 2018 | Yes | Partial Yes | Yes | Partial Yes | Yes | Yes | Yes | Yes | Partial Yes | Yes | No MA | No MA | Yes | Yes | No | Yes | Moderate |
| Liangruenrom 2019 | Yes | Partial Yes | Yes | Partial Yes | Yes | Yes | Yes | Partial Yes | Partial Yes | Yes | No MA | No MA | Yes | Yes | No | Yes | Moderate |
| Lindsay Smith 2017 | Yes | Partial Yes | Yes | Partial Yes | Yes | Yes | Yes | Partial Yes | Partial Yes | No | No MA | No MA | Yes | No | No | Yes | Moderate |
| Lorenc 2008 | Yes | Partial Yes | Yes | Partial Yes | Yes | Yes | Partial Yes | Partial Yes | Partial Yes | No | No MA | No MA | Yes | Yes | No | Yes | Moderate |
| Maitland 2013 | Yes | Partial Yes | Yes | Partial Yes | Yes | Yes | Yes | Partial Yes | Partial Yes | Yes | No MA | No MA | Yes | Yes | No | Yes | Moderate |
| Mendonça 2014 | Yes | No | Yes | Yes | Yes | Yes | Partial Yes | No | Yes | Partial Yes | No MA | No MA | Yes | Yes | No | Yes | Low |
| Olekszechein 2016 | Yes | Partial Yes | Yes | Partial Yes | No | No | Partial Yes | Partial Yes | No | No | No MA | No MA | No | N | No | Yes | Low |
| Pan 2021 | Yes | Partial Yes | Yes | Yes | Yes | No | Partial Yes | Partial Yes | Yes | Yes | Yes | Yes | Yes | Yes | Yes | Yes | High |
| Pollard 2017 | Yes | Partial Yes | Yes | Partial Yes | Yes | Yes | Yes | Partial Yes | Partial Yes | Yes | Yes | Yes | Yes | Yes | Yes | Yes | High |
| Pont 2009 | Yes | Partial Yes | Yes | Partial Yes | Yes | Yes | Yes | No | Yes | No | No MA | No MA | Yes | No | No | Yes | Moderate |
| Rhodes 2013 | Yes | Partial Yes | Yes | Partial Yes | Yes | Yes | Yes | Partial Yes | Partial Yes | Yes | No MA | No MA | Yes | Yes | No | Yes | Moderate |
| Rhodes 2020 | Yes | Partial Yes | No | Partial Yes | Yes | Yes | Yes | Partial Yes | Yes | Yes | No  MA | No MA | Yes | Yes | No | Yes | Moderate |
| Ridgers 2012 | Yes | Partial Yes | Yes | Partial Yes | Yes | Yes | Yes | Partial Yes | No | Yes | No MA | No MA | Yes | Yes | No | Yes | Moderate |
| Rothman 2018 | Yes | Partial Yes | Yes | Partial Yes | Yes | Yes | Yes | Partial Yes | Partial Yes | Yes | No MA | No MA | Yes | No | No | Yes | Moderate |
| Salvo 2018 | No | No | Yes | Partial Yes | No | No | No | Partial Yes | No | Yes | No MA | No MA | Yes | Yes | No | Yes | Low |
| Scarapicchia 2017 | Yes | Partial Yes | No | Partial Yes | No | No | Yes | Partial Yes | Yes | No | Yes | Yes | Yes | Yes | Yes | Yes | Moderate |
| Smith 2017 | Yes | Partial Yes | Yes | Partial Yes | Yes | Yes | Yes | Partial Yes | Partial Yes | Yes | No MA | No MA | Yes | Yes | No | Yes | Moderate |
| Stanley 2012 | Yes | Partial Yes | Yes | Partial Yes | Yes | Yes | Yes | Partial Yes | Partial Yes | Yes | No MA | No MA | Yes | Yes | No | Yes | Moderate |
| Stappers 2018 | Yes | Partial Yes | Yes | Partial Yes | Yes | Yes | Yes | Partial Yes | Yes | No | No MA | No MA | Yes | Yes | No | Yes | Low |
| Tovar 2018 | Yes | Partial Yes | Yes | Partial Yes | Yes | Yes | Yes | Partial Yes | Partial Yes | Yes | No MA | No MA | Yes | Yes | No | Yes | Moderate |
| Van Cauwenberg 2011 | Yes | Partial Yes | Yes | Partial Yes | No | No | Yes | Partial Yes | Partial Yes | Yes | No MA | No MA | Yes | No | No | Yes | Moderate |
| Van Cauwenberg 2018 | Yes | Yes | Partial Yes | Yes | Yes | Yes | Partial Yes | Partial Yes | Partial Yes | No | Yes | Yes | No | No | Yes | Yes | Low |
| Van Hecke 2018 | Yes | Partial Yes | Yes | Partial Yes | Yes | Yes | Yes | Partial Yes | Yes | Yes | No MA | No MA | Yes | Yes | No | Yes | Moderate |
| Van Holle 2012 | Yes | Partial Yes | Yes | Partial Yes | Yes | Yes | Partial Yes | Partial Yes | Partial Yes | Yes | No MA | No MA | Yes | No | No | Yes | Moderate |
| Xiao 2019 | Yes | Partial Yes | Yes | Partial Yes | Yes | Yes | Yes | No | Partial Yes | No | Yes | Yes | Yes | Yes | Yes | Yes | Moderate |
| Yarmohammadi 2019 | Yes | Partial Yes | Yes | Partial Yes | Yes | Yes | Yes | Partial Yes | Yes | Yes | No MA | No MA | Yes | Yes | No | Yes | Moderate |
| Zhang 2019 | Yes | Partial Yes | Yes | Partial Yes | Yes | Yes | Yes | Partial Yes | Yes | Yes | No MA | No MA | Yes | Yes | No | Yes | Moderate |

Items: (1) Did the research questions and inclusion criteria for the review include the components of PICO?; (2) Did the report of the review contain an explicit statement that the review methods were established prior to the conduct of the review and did the report justify any significant deviations from the protocol?; (3) Did the review authors explain their selection of the study designs for inclusion in the review?; (4) Did the review authors use a comprehensive literature search strategy?; (5) Did the review authors perform study selection in duplicate?; (6) Did the review authors perform data extraction in duplicate?; (7) Did the review authors provide a list of excluded studies and justify the exclusions?; (8) Did the review authors describe the included studies in adequate detail?; (9) Did the review authors use a satisfactory technique for assessing the RoB in individual studies that were included in the review?; (10) Did the review authors report on the sources of funding for the studies included in the review?; (11) If MAs was performed did the review authors use appropriate methods for statistical combination of results?; (12) If MAs was performed, did the review authors assess the potential impact of RoB in individual studies on the results of the MAs or other evidence synthesis?; (13) Did the review authors account for RoB in individual studies when interpreting/ discussing the results of the review?; (14) Did the review authors provide a satisfactory explanation for, and discussion of, any heterogeneity observed in the results of the review?; (15) If they performed quantitative synthesis did the review authors carry out an adequate investigation of publication bias and discuss its likely impact on the results of the review?; (16) Did the review authors report any potential sources of conflict of interest, including any funding they received for conducting the review?; AMSTAR, Assessing the Methodological Quality of Systematic Reviews; MA, meta-analysis.
